# Improvements in Memory after Medial Septum Stimulation Are Associated with Changes in Hippocampal Cholinergic Activity and Neurogenesis

**DOI:** 10.1155/2014/568587

**Published:** 2014-07-02

**Authors:** Da Un Jeong, Ji Eun Lee, Sung Eun Lee, Won Seok Chang, Sung June Kim, Jin Woo Chang

**Affiliations:** ^1^Brain Korea 21 PLUS Project for Medical Science and Brain Research Institute, Yonsei University College of Medicine, Seoul 120-752, Republic of Korea; ^2^Brain Korea 21 PLUS Project, Department of Electrical and Computer Engineering, College of Engineering, Seoul National University, Seoul 151-744, Republic of Korea; ^3^Department of Electrical and Computer Engineering, College of Engineering, Seoul National University, Seoul 151-744, Republic of Korea; ^4^Inter-University Semiconductor Research Center, Seoul National University, Seoul 151-744, Republic of Korea; ^5^Department of Neurosurgery, Severance Hospital, Yonsei University College of Medicine, Seoul 120-752, Republic of Korea

## Abstract

Deep brain stimulation (DBS) has been found to have therapeutic effects in patients with dementia, but DBS mechanisms remain elusive. To provide evidence for the effectiveness of DBS as a treatment for dementia, we performed DBS in a rat model of dementia with intracerebroventricular administration of 192 IgG-saporins. We utilized four groups of rats, group 1, unlesioned control; group 2, cholinergic lesion; group 3, cholinergic lesion plus medial septum (MS) electrode implantation (sham stimulation); group 4, cholinergic lesions plus MS electrode implantation and stimulation. During the probe test in the water maze, performance of the lesion group decreased for measures of time spent and the number of swim crossings over the previous platform location. Interestingly, the stimulation group showed an equivalent performance to the normal group on all measures. And these are partially reversed by the electrode implantation. Acetylcholinesterase activity in the hippocampus was decreased in lesion and implantation groups, whereas activity in the stimulation group was not different from the normal group. Hippocampal neurogenesis was increased in the stimulation group. Our results revealed that DBS of MS restores spatial memory after damage to cholinergic neurons. This effect is associated with an increase in hippocampal cholinergic activity and neurogenesis.

## 1. Introduction

It is widely accepted that brain function can be modulated by electrical stimulation of focal brain structures; furthermore, that electrical stimulation may possibly be used to treat patients with brain dysfunction. In particular, deep brain stimulation (DBS) has been used to treat various types of movement disorders and psychiatric disorders [1–3]. Recently, DBS of memory-associated brain structures were tested as a possible treatment for Alzheimer's-type dementia, with some studies providing promising results. For example, some authors report that hypothalamic stimulation modulates limbic activity and improves certain memory functions [4], and others report that DBS of the nucleus basalis magnocellularis (NBM) improves cognitive functioning in patients with Parkinson's disease-related dementia [5]. Furthermore, stimulation of the entorhinal region was found to enhance memory for spatial information when applied during learning [6]. In animal studies, high frequency stimulation activates specific amino acids in the hippocampus that may be involved in the enhancement of short-term memory formation [7]. Also, formation of water maze memory is facilitated after bilateral stimulation of the entorhinal cortex [8].

Clinical evidence obtained from patients with dementia or other neurological disorders shows that DBS could be used as a tool to enhance memory function. However, this evidence is not strong, as the clinical trials were not randomized and case-controlled or did not employ large sample sizes. Therefore, disease-specific animal experiments are necessary to advance the clinical application of DBS. There are varied results due to the administration of 192 IgG-saporin [9–14]. We made the experiment with a rat model of dementia by intracerebroventricular (ICV) 192 IgG-saporin injections. And we found that spatial memory is profoundly impaired in this model [15]. Here, we used the 192 IgG-saporin rat model of dementia to confirm the effects of DBS on spatial memory function.

Degeneration of cholinergic basal forebrain neurons, including the medial septum (MS), is a common feature of Alzheimer's disease and vascular dementia and has been correlated with cognitive decline [16–19]. Projections from the MS to the hippocampus consist of more than three types of fibers, including cholinergic, GABAergic, and glutamatergic fibers [20–22] and are the primary source of cholinergic input to the hippocampus [23, 24]. Furthermore, the MS is reported to regulate hippocampal activity through acetylcholine, GABA, and glutamate [25–28].

DBS therapy for dementia has not yet established the most effective stimulation site and parameter. In general, high frequency stimulation leads to long term potentiation, and low frequency stimulation induces long term depression [29]. However, recent reports showed that long term potentiation could be induced by low frequency stimulation [30, 31]. Some studies evaluating the hippocampal effect by the MS stimulation are mostly divided into low frequency (<10 Hz) or high frequency (>100 Hz) stimulation [32–34]. Therefore, we stimulated the MS with midfrequency (60 Hz) stimulation trains to evaluate the therapeutic potential of this frequency range for reversing spatial memory impairments induced by the loss of cholinergic neurons.

## 2. Materials and Methods

### 2.1. Animals

The present study was conducted according to the guidelines for the care and use of laboratory animals of the NATIONAL RESEARCH COUNCIL, USA. Rats were housed one to three per cage in a temperature- and humidity-controlled room, and all rats had free access to food and water with a 12-hour light/12-hour dark cycle.

Fifty male Sprague Dawley rats (200–250 g) were randomly assigned to one of the following groups. Rats in the normal (*n* = 11) group had no surgical procedure. Rats in the lesion (*n* = 15) group had ICV administration of 192 IgG-saporin. Rats in the implantation (*n* = 14) group had ICV administration of 192 IgG-saporin and implantation of an electrode in the MS. Rats in the stimulation (*n* = 10) group had ICV administration of 192 IgG-saporin and electrical stimulation of the MS.

### 2.2. Surgical Procedure

Thirty-nine rats were anesthetized with a mixture of ketamine (75 mg/kg), acepromazine (0.75 mg/kg), and rompun (4 mg/kg) and secured in a stereotaxic frame. After scalp incision, rats were injected bilaterally with 8 *μ*L of 192 IgG-saporin (0.63 *μ*g/*μ*L, Chemion, Temecula, CA) into ICV following coordinates relating to Bregma: AP: −0.8 mm, ML: ±1.2 mm, and DV: −3.4 mm. The solutions were delivered at a rate of 1 *μ*L/min. The syringe was left in place for 5 min after injection.

After the administration of 192 IgG-saporin, 24 rats had an additional procedure for electrode implantation. A hole was drilled in the skull at the level of the MS, and a tungsten electrode was implanted in the MS (AP + 0.6 mm, ML 0.1 mm, and DV − 6 mm). The stimulation electrode was fixed with dental cement (Long Dental Manufacturing, Wheeling, IL). This group was further divided into two groups, with 12 rats originally in the implantation group and 12 rats originally in the simulation group. However, two rats in the stimulation group were not stimulated due to a problem with the wire. Therefore, the implantation group consisted of 14 rats, and the stimulation group consisted of 10 rats.

### 2.3. Stimulation Electrode and Parameters

A tungsten stimulation electrode (200 *μ*m diameter, 5 *μ*m parylene coating) was inserted into the MS. The tip of electrode was tapered by electrochemical etching to reduce damage to the target area electrical stimulation consisted of pulses (120 *μ*s duration, 50 *μ*A) delivered at 60 Hz. Stimulation parameters were monitored in real time at the beginning and end of stimulation with an oscilloscope (HDS 1022M, Owon, Korea). Rats were stimulated daily beginning a week after surgery until the end of behavioral testing (5 consecutive hours per day, every day for 2 weeks). Stimulation was delivered after the daily water maze training session.

### 2.4. Behavioral Test: Morris Water Maze

Two weeks after surgery, rats were trained in the Morris water maze as previously described [15]. Training consisted of four trials per day for 5 days with the platform in a fixed position. For each training trial, the rat was placed into the pool at one of four semirandomly chosen starting points and given 60 s to reach the platform. Any rat that did not reach the platform within 60 s was led to the platform by the experimenter and allowed to remain on the platform for 10 s. After 48 hours from the final training trial, the rats were given a probe trial lasting 60 s, during which the platform was removed from the pool. Swim paths were recorded using a video tracking system. During training trials, swim distance, latency to reach the platform, and swim speed were measured. During the probe trial, swim distance, swim speed, swim time in each quadrant, the time spent in the platform zone, and the number of platform crossings were also measured.

### 2.5. Histological Evaluation

Immediately after behavioral testing (probe test), 8 out of 11 rats from the normal group, 8 out of 15 rats from the lesion group, 8 out of 14 rats from the implantation group, and 6 out of 10 rats from the stimulation group were anesthetized with a mixture of ketamine, acepromazine, and rompun and perfused with normal saline and cold 4% paraformaldehyde. Brains were removed, postfixed, and transferred to 30% sucrose for 4 days. The brains were sectioned into 30 *μ*m sections using a freezing microtome and stored in a cryoprotectant solution (0.1 M phosphate buffer (pH 7.2), 30% sucrose, 1% polyvinylpyrrolidone, and 30% ethylene glycol) at −20°C. Anatomical landmarks from a stereotaxic atlas [35] were used to localize the MS and hippocampus.

Floating sections were used to detect location of electrode, cholinergic cells, and neurogenesis. Cresyl violet staining was performed to confirm the location of electrode. To perform immunohistochemistry, sections were incubated in 0.3% H_2_O_2_ for 30 min to inactivate endogenous peroxidase activity. They were blocked with 5% normal serum and incubated with polyclonal antibodies against choline acetyltransferase (1 : 200, ChAT, Chemicon, Temecula, CA) or doublecortin (1 : 200, Santa Cruz Biotechnology Inc., Santa Cruz, CA) overnight at 4°C. The sections were incubated with biotinylated secondary antibodies, followed by the avidin-biotin complex method (ABC Elite, Vector Labs, Burlingame, CA). They were visualized with diaminobenzidine (DAB) using a DAB substrate kit (Thermo, Fremont, CA).

### 2.6. Acetylcholinesterase (AChE) Assay

The remaining rats (3 out of 11 rats from the normal group, 7 out of 15 rats from the lesion group, 6 out of 14 rats from the implantation group, and 4 out of 10 rats from the stimulation group) were anesthetized with a mixture of ketamine, acepromazine, and rompun and decapitated with a guillotine. The brains were quickly removed to acquire protein for AChE assay. The MS and hippocampus were dissected with fine forceps from 1 mm coronal brain slices. The samples were homogenized in lysis buffer (Intron, Seongnam, Korea) and placed in ice for 30 min. The samples were centrifuged for 20 min at 12,000 rpm, and the protein in supernatant was measured using the bicinchoninic acid protein assay reagent kit (Pierce, Rockford, IL). The protein samples were stored at −70°C. The enzymatic activity of AChE was determined using the method of Ellman et al. [36] with some modifications as previously described [15]. Briefly, 20 *μ*L triplicate samples were mixed with the reaction mixture (0.2 mM dithiobisnitrobenzoic acid (Sigma, St. Louis, MO), 0.56 mM acetylthiocholine iodide (Sigma, St. Louis, MO), 10 *μ*M tetraisopropyl pyrophosphoramide (Sigma, St. Louis, MO), and 39 mM phosphate buffer, pH 7.2) at 37°C. After 30 min, the optical density was measured at 405 nm.

### 2.7. Data Analysis

Indices of water maze probe test were expressed as a percentage of the values of the normal group. Doublecortin immunopositive cells were counted in 10 coronal sections per group, located 3.0 to 3.6 mm posterior to bregma. The number of doublecortin immunopositive cells was presented as mean ± standard error of the mean (SEM). One-way analysis of variance was used for overall analysis of experiments except training trials, followed by a least significant difference test or Tukey honestly significant difference test as post hoc tests at each time point. *P* values less than 0.05 were considered statistically significant. Water maze training trials were analysed with Linear Mixed Model. Statistical analyses were performed using PASW (version 18; SPSS Inc., Chicago, IL) and SAS version 9.2 (SAS Institute Inc., Cary, NC, USA).

## 3. Results

### 3.1. Spatial Memory Testing

**Figure 1:**
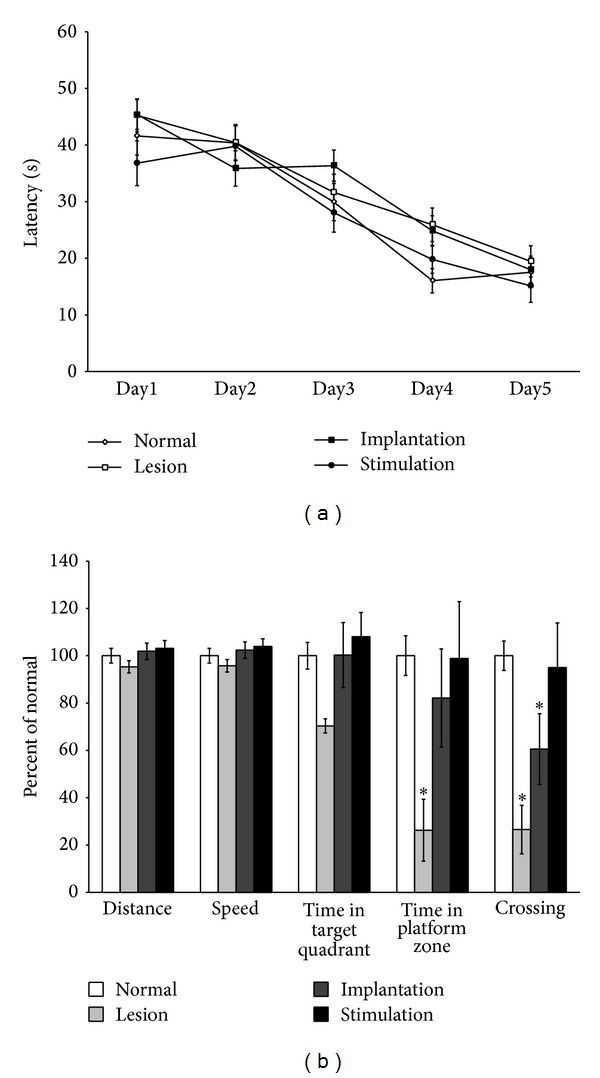
Effect of MS-DBS on spatial memory. Latency indicates the time required for the rat to find the escape platform during training trails. During training trails, all groups gradually acquired the location of the platform (a). After a delay of two days, spatial memory was improved by MS-DBS (b). Time spent in the platform zone (*P* < 0.05) and number of crossings (*P* < 0.005) was significantly different between lesion and normal groups. However, the stimulation group did not differ from the normal group. There was no disruption of motor function in any group. Data are shown as mean ± SEM (a). Indices are expressed as the percentage of normal group values (b). MS: medial septum; DBS: deep brain stimulation: Dist: distance.

The results of water maze training are shown in Figure 1(a). The latencies of the first day were decreased under the 20 seconds on the final day of the training trails. From the first day to the fifth day, the differences among the days were statistically significant (*P* < 0.0001) regardless of group. However, there was no significant difference among the groups depending on the time passage (*P* = 0.3897). Taken together, these data show that latency to reach the platform declined progressively across training days for all groups, indicating progressive learning of the hidden platform location. Water maze probe test indices were expressed as a percentage of values for the normal group (Figure 1(b)). The lesion, implantation, and stimulation groups showed no differences from the normal group in motor-related behavior, evidenced by similar swim distances and speeds. These findings suggest no effect of cholinergic lesion, electrode implantation, or electrical stimulation on motor function. However, the amount of time that the lesion group spent in the target quadrant was decreased to 70% of normal group values. Also, the amount of time that the lesion group spent in the platform zone was significantly decreased to 26% of normal group values (*P* = 0.006), whereas it was only decreased to 70% (*P* = 0.472) and 98% (*P* = 0.965) for the implantation and stimulation groups, respectively. Moreover, the number of platform crossings was significantly reduced to 27% (*P* < 0.001) and 61% (*P* = 0.039) for the lesion and implantation groups, respectively, whereas it was only decreased to 95% for the stimulation group (*P* = 0.805).

### 3.2. Histological Evaluations

**Figure 2:**
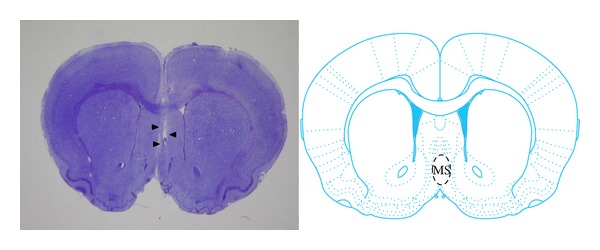
Cresyl violet stained coronal section and slide of atlas [35] at MS level. Arrow heads show the tract of the electrode in the MS.

The location of stimulating electrodes in the MS was confirmed with cresyl violet staining (Figure 2).

**Figure 3:**
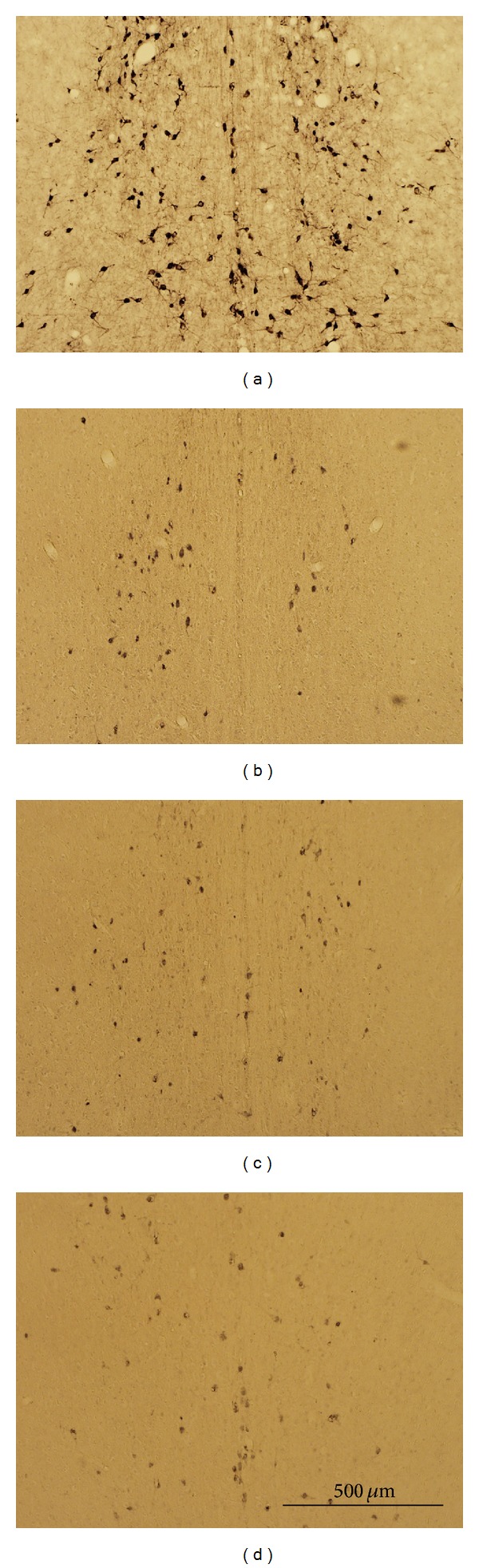
Representative pictures showing the effects of the cholinergic lesion on the basal forebrain. The normal group had numerous ChAT immunopositive neurons in the MS (a). The lesion (b), implantation (c), and stimulation (d) groups displayed a loss of cholinergic neurons in the MS. Scale bar represents 500 *μ*m. ChAT: choline acetyltransferase; MS: medial septum.

Intraventricular 192 IgG-saproin injections produced denervation of ChAT immunopositive neurons in the MS, which is thought to be part of the basal forebrain complex (Figure 3). The ChAT immunopositive neurons in normal rats were evenly distributed in the MS, and the structure of the cell bodies and dendrites were wholly intact as shown in Figure 3(a). In contrast, lesion, implantation, and stimulation groups, which were injected with 192 IgG-saporin, showed noticeable damage of cell body and dendrite structures.

**Figure 4:**
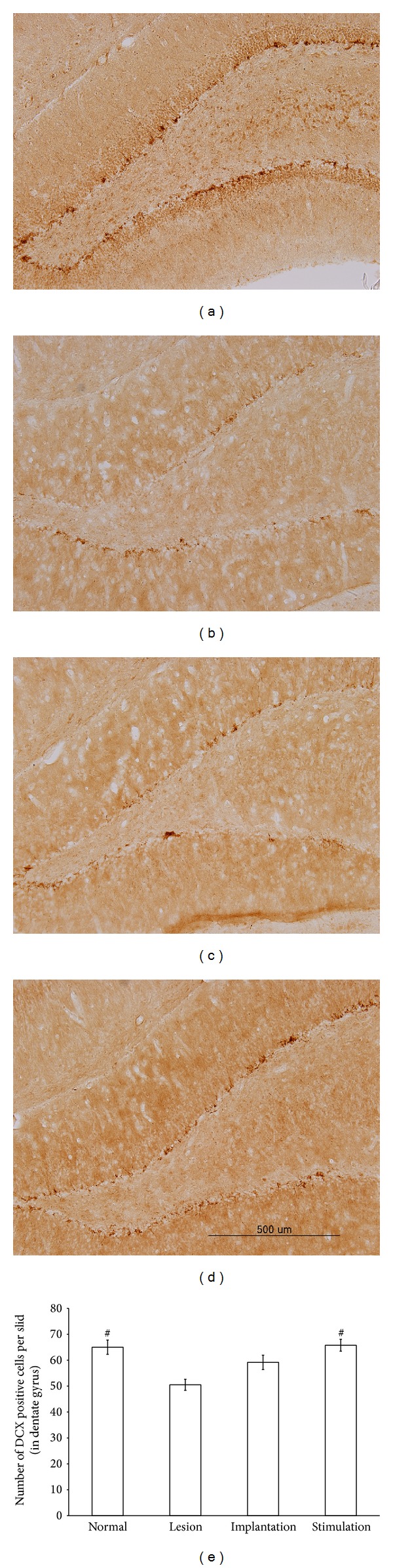
Effects of MS-DBS on adult hippocampal neurogenesis revealed by doublecortin immunohistochemistry. Representative pictures show the effects of basal forebrain cholinergic deficits and MS-DBS on hippocampal neurogenesis (a–d). Many doublecortin immunopositive cells were observed in the normal group (a). However, the number of these cells was decreased in the lesion (b) and implantation (c) groups, in which basal forebrain cholinergic neurons were damaged, but not in the stimulation group (d). After counting the immunopositive cells (e), we found that the number of cells in the normal group was significantly different from that in the lesion group (*P* < 0.05). However, there was no difference between the lesion group and the implantation group, which was only implanted with an electrode in the MS. The number of doublecortin immunopositive cells was significantly increased in the stimulation group (*P* < 0.05), which had damaged basal forebrain cholinergic neurons and received electrical stimulation of the MS.

We quantified the number of hippocampal cells containing doublecortin, which is expressed in various neurogenesis stages from the differentiation phase to the axonal and dendrite targeting phase, using immunohistochemistry (Figure 4). The lesion group showed a significant decline in numbers of doublecortin immunopositive cells to 77.6% of normal group values (*P* = 0.002), which confirms the effect of damaged basal forebrain cholinergic neuron on hippocampal neurogenesis. The implantation group did not show a significant difference from the lesion group (*P* = 0.142). However, the stimulation group showed a significant increase in the number of doublecortin immunopositive cells compared with the lesion group (*P* = 0.002).

### 3.3. AChE Assay

**Figure 5:**
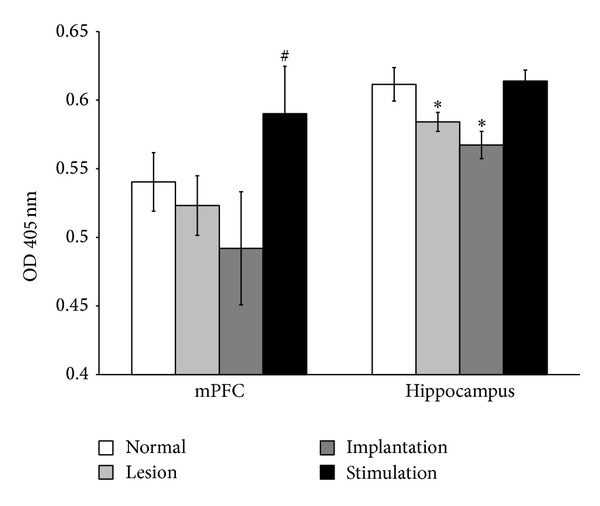
Effect of MS-DBS on AChE activity. In the prefrontal cortex, AChE activity of the stimulation group was significantly increased more than that in implantation group (*P* < 0.05). Hippocampal AChE activity in the lesion (*P* < 0.05) and implantation (*P* < 0.005) groups was significantly less than that in the normal group. However, AChE activity in the stimulation group was equivalent to that in the normal group. The AChE activity was expressed as the optical density of the colorimetric reading at 405 nm. Values are mean ± SEM. OD: optical density.

In the medial prefrontal cortex, AChE activity was declined in the lesion and implantation groups (Figure 5), although there were no statistically significant differences from the normal group. AChE activity in the stimulation group was higher than that in the normal group, and it was more significantly increased than that in the implantation group (*P* = 0.028). There was a statistically significant decline in hippocampal AChE activity in the lesion group (*P* = 0.045) and implantation group (*P* = 0.001) compared with the normal group. Interestingly, hippocampal AChE activity in the stimulation group was similar to that in the normal group and it is more significantly increased than that in the lesion (*P* = 0.038) and implantation groups (*P* = 0.001).

## 4. Discussion

Because medications for dementia have limitations, such as side effects or temporary efficacy, the study of alternative therapies is needed. DBS has been approved by the US Food and Drug Administration and can safely be used for movement disorders like Parkinson's disease or tremor. Recently, the possibility that DBS can enhance memory function has been reported in some clinical cases and experimental studies [4–8]. However, the mechanisms of DBS are unclear, and effective stimulation parameters, such as duration and location, are still not well defined. Therefore, experimental studies are needed.

Longer durations of MS-DBS might provide greater therapeutic effects than shorter durations of stimulation. Therefore, we stimulated the MS starting 1 week before the behavioral test and continued stimulating until the end of the test. Laxton et al. [37] reported possible improvements in cognition and slowing of the rate of cognitive decline by continuous fornix/hypothalamus DBS for 12 months. Compared to baseline, an increase in brain metabolism in the temporal and parietal cortical regions was observed 1 month after DBS and was sustained for 12 months. We confirmed that motor function was unaltered in the stimulation group, which received electrical stimulation for 2 weeks.

Although we do not know which stage of memory processing was affected by MS-DBS, long term spatial memory was improved. An interesting finding in this study was that the impairment in spatial memory by 192 IgG-saporin was rescued by MS-DBS. Therefore, we propose that 60 Hz MS-DBS has positive effects on spatial memory.

It has been reported that ICV injection of 192 IgG-saporin damaged both cholinergic basal forebrain neurons and Purkinje cell in the cerebellum [38]. Also, Cerebellar Purkinje cell loss in AD patients has been reported. Because of the similarity in the loss of Purkinje cell [39, 40], using of 192 IgG-saporin is not a big problem for making a dementia model. When 192 IgG-saporin is injected into the ICV rather than direct administration in MS, cholinergic cells have higher survival rate. In this study, we administrated 192 IgG-saporin into the ICV. And averagely 35% of the cholinergic cells were intact in the MS. Our finding of residual ChAT immunopositive cells and AChE activity indicates that basal forebrain cholinergic neurons were not completely damaged by administration of intraventricular 192 IgG-saporin. We believe that MS-DBS influenced the activity of the hippocampus via projections from the MS through remaining cholinergic neurons despite injection of 192 IgG-saporin.

A single shock or brief tetanic stimulation of the MS was found to sharply enhance population spikes evoked in the hippocampal CA1 pyramidal cell layer, and a comparable facilitation of population spikes was produced by microapplication of acetylcholine at the same site [41]. Both cholinergic and GABAergic mechanisms have been proposed to explain this effect. MS facilitation of hippocampal activity is mediated by inhibition of inhibitory interneurons [42]. Cholinergic- and GABAergic-mediated septal drive plays a role in the tuning of signal conversion within the hippocampus [43]. Low-frequency stimulation of the MS and commissural fibers induces NMDA-dependent, long-lasting potentiation of hippocampal synapses [30]. Electrical stimulation of the MS could possibly affect these systems, although we did not investigate GABAergic or glutamatergic changes in this study. Moreover, the increase in hippocampal acetylcholine by MS-DBS could affect hippocampal theta rhythm and spatial memory. Considerable research has demonstrated correlations between hippocampal theta rhythm and learning and memory [44–46], and positive correlations between hippocampal acetylcholine and theta rhythm have also been reported [47–49].

The other potential mechanism through which MS-DBS could enhance spatial memory is via hippocampal neurogenesis. Adult hippocampal neurogenesis is restricted to the subgranular zone of the dentate gyrus (DG), and new neurons continue to be generated throughout the lifespan. Hippocampal neurogenesis is thought to be associated with hippocampus-dependent memory. Knockdown of adult hippocampal neurogenesis impairs spatial memory [50], and treatments that disrupt hippocampal neurogenesis impair hippocampus-dependent memory [51, 52]. Adult hippocampal neurogenesis is thought to consist of several developmental processes [53]. Doublecortin (DCX) is a protein that promotes microtubule polymerization and can serve as a marker of adult neurogenesis during the stages of late mitotic neuronal precursors and early postmitotic neurons [54, 55]. DCX is expressed specifically in newly generated neurons. DCX is not expressed in GFPA-expressing astrocytes. Also the study shows absence of DCX expression during neuronal regeneration or lesion induced gliogenesis [56]. We confirmed that a deficiency of basal forebrain cholinergic neurons reduces the number of DCX immunopositive cells in the subgranular zone of the DG. This result supports previous findings that damage of basal forebrain cholinergic neurons decreases neurogenesis in the granule cell layer of the DG and increases the number of apoptotic cells [57–59]. Interestingly, Stone et al. reported that electrical stimulation of the entorhinal cortex promotes proliferation in the DG and the integration of these neurons into hippocampal circuits supporting spatial memory [8]. In this study, stimulation of the MS after cholinergic damage recovers the number of DCX immunopositive cells, suggesting that MS-DBS promotes neurogenesis in the DG, which, in turn, may promote hippocampus-dependent learning and memory. We speculate that MS-DBS may increase hippocampal acetylcholine and thereby promote hippocampal neurogenesis. Cholinergic activation increases proliferation of hippocampal neural stem cells and enhances the survival of newborn neurons [60, 61].

Unexpectedly, the behavioral test deficits of lesioned rats seen during the probe trials appear to be partially reversed by the electrode implantation, even in the absence of electrical stimulation. Insertion effect is common phenomenon after deep brain stimulation surgery for Parkinson disease or neuropathic pain patients [62, 63]. After the insertion of electrode into the subthalamic nucleus for PD patients, glucose metabolism has been changed in the absence of stimulation [64]. In the unstimulated hippocampus, enzyme activity is changed in the narrow area surrounding the electrode [65]. In animal study, it has been reported that the different thickness of electrode induced a various regional neuroinflammation and recognition deficits [66]. This different effect on memory from our results could be caused by the thickness of used electrode (280, 150 *μ*m versus 200 *μ*m) and the site of implantation (subthalamic nucleus versus MS). Because the insertional effects are still unclear, the relevant studies are needed in the future.

In conclusion, we found that MS-DBS increases hippocampal acetylcholine and neurogenesis and restore spatial memory from cholinergic malfunction due to basal forebrain cholinergic deficiency. Although we do not know which stage of memory processing is affected by MS-DBS, our study is important in that it confirms the therapeutic effect of MS-DBS.

## References

[1] Larson PS (2008). Deep brain stimulation for psychiatric disorders. *Neurotherapeutics*.

[2] Lyons MK (2011). Deep brain stimulation: current and future clinical applications. *Mayo Clinic Proceedings*.

[3] Wichmann T, DeLong MR (2006). Deep brain stimulation for neurologic and neuropsychiatric disorders. *Neuron*.

[4] Hamani C, McAndrews MP, Cohn M (2008). Memory enhancement induced by hypothalamic/fornix deep brain stimulation. *Annals of Neurology*.

[5] Freund H, Kuhn J, Lenartz D (2009). Cognitive functions in a patient with parkinson-dementia syndrome undergoing deep brain stimulation. *Archives of Neurology*.

[6] Suthana N, Haneef Z, Stern J (2012). Memory enhancement and deep-brain stimulation of the entorhinal area. *New England Journal of Medicine*.

[7] Luna-Munguía H, Meneses A, Peña-Ortega F, Gaona A, Rocha L (2012). Effects of hippocampal high-frequency electrical stimulation in memory formation and their association with amino acid tissue content and release in normal rats. *Hippocampus*.

[8] Stone SSD, Teixeira CM, DeVito LM (2011). Stimulation of entorhinal cortex promotes adult neurogenesis and facilitates spatial memory. *Journal of Neuroscience*.

[9] Leanza G, Nilsson OG, Wiley RG, Bjorklund A (1995). Selective lesioning of the basal forebrain cholinergic system by intraventricular 192 IgG-saporin: Behavioural, biochemical and stereological studies in the rat. *European Journal of Neuroscience*.

[10] Chappell J, McMahan R, Chiba A, Gallagher M (1998). A re-examination of the role of basal forebrain cholinergic neurons in spatial working memory. *Neuropharmacology*.

[11] Dornan WA, McCampbell AR, Tinkler GP (1996). Comparison of site-specific injections into the basal forebrain on water maze and radial arm maze performance in the male rat after immunolesioning with 192 IgG saporin. *Behavioural Brain Research*.

[12] Torres EM, Perry TA, Blokland A (1994). Behavioural, histochemical and biochemical consequences of selective immunolesions in discrete regions of the basal forebrain cholinergic system. *Neuroscience*.

[13] Walsh TJ, Kelly RM, Dougherty KD, Stackman RW, Wiley RG, Kutscher CL (1995). Behavioral and neurobiological alterations induced by the immunotoxin 192-IgG-saporin: cholinergic and non-cholinergic effects following i.c.v. injection. *Brain Research*.

[14] Chang Q, Gold PE (2004). Impaired and spared cholinergic functions in the hippocampus after lesions of the medial septum/vertical limb of the diagonal band with 192 IgG-saporin. *Hippocampus*.

[15] Jeong DU, Chang WS, Hwang YS, Lee D, Chang JW (2011). Decrease of GABAergic markers and arc protein expression in the frontal cortex by intraventricular 192 IgG-saporin. *Dementia and Geriatric Cognitive Disorders*.

[16] Bierer LM, Haroutunian V, Gabriel S (1995). Neurochemical correlates of dementia severity in Alzheimer's disease: relative importance of the cholinergic deficits. *Journal of Neurochemistry*.

[17] Kása P, Rakonczay Z, Gulya K (1997). The cholinergic system in alzheimer's disease. *Progress in Neurobiology*.

[18] Toledano A, Alvarez MI (2004). Lesions and dysfunctions of the nucleus basalis as Alzheimer's disease models: general and critical overview and analysis of the long-term changes in several excitotoxic models. *Current Alzheimer Research*.

[19] Baxter MG, Chiba AA (1999). Cognitive functions of the basal forebrain. *Current Opinion in Neurobiology*.

[20] Dudar JD (1977). The role of the septal nuclei in the release of acetylcholine from the rabbit cerebral cortex and dorsal hippocampus and the effect of atropine. *Brain Research*.

[21] Sotty F, Danik M, Manseau F, Laplante F, Quirion R, Williams S (2003). Distinct electrophysiological properties of glutamatergic, cholinergic and GABAergic rat septohippocampal neurons: novel implications for hippocampal rhythmicity. *The Journal of Physiology*.

[22] Price DL, Koliatsos VE, Clatterbuck RC (1993). Cholinergic systems: human diseases, animal models, and prospects for therapy. *Progress in Brain Research*.

[23] Lewis PR, Shute CC, Silver A (1967). Confirmation from choline acetylase analyses of a massive cholinergic innervation to the rat hippocampus.. *Journal of Physiology*.

[24] Lynch G, Rose G, Gall C (1977). Anatomical and functional aspects of the septo-hippocampal projections. *Ciba Foundation Symposium*.

[25] Jouvenceau A, Dutar P, Billard JM (1998). Alteration of NMDA receptor-mediated synaptic responses in CA1 area of the aged rat hippocampus: contribution of GABAergic and cholinergic deficits. *Hippocampus*.

[26] Kanju PM, Parameshwaran K, Sims-Robinson C (2012). Selective cholinergic depletion in medial septum leads to impaired long term potentiation and glutamatergic synaptic currents in the hippocampus. *PLoS ONE*.

[27] Pang KCH, Jiao X, Sinha S, Beck KD, Servatius RJ (2011). Damage of GABAergic neurons in the medial septum impairs spatial working memory and extinction of active avoidance: effects on proactive interference. *Hippocampus*.

[28] Jouvenceau A, Billard JM, Lamour Y, Dutar P (1997). Potentiation of glutamatergic EPSPs in rat CA1 hippocampal neurons after selective cholinergic denervation by 192 IgG-saporin. *Synapse*.

[29] Caporale N, Dan Y (2008). Spike timing-dependent plasticity: a Hebbian learning rule. *Annual Review of Neuroscience*.

[30] Habib D, Dringenberg HC (2009). Alternating low frequency stimulation of medial septal and commissural fibers induces NMDA-dependent, long-lasting potentiation of hippocampal synapses in urethane-anesthetized Rats. *Hippocampus*.

[31] Huang Y, Kandel ER (2007). Low-frequency stimulation induces a pathway-specific late phase of LTP in the amygdala that is mediated by PKA and dependent on protein synthesis. *Learning and Memory*.

[32] Bland BH, Bird J, Jackson J, Natsume K (2006). Medial septal modulation of the ascending brainstem hippocampal synchronizing pathways in the freely moving rat. *Hippocampus*.

[33] Galey D, Destrade C, Jaffard R (1994). Relationships between septo-hippocampal cholinergic activation and the improvement of long-term retention produced by medial septal electrical stimulation in two inbred strains of mice. *Behavioural Brain Research*.

[34] Habib D, Dringenberg HC (2010). Surprising similarity between mechanisms mediating low (1 Hz)-and high (100 Hz)-induced long-lasting synaptic potentiation in CA1 of the intact hippocampus. *Neuroscience*.

[35] Paxions G, Watson C (2007). *The Rat Brain in Stereotaxic Coordinates*.

[36] Ellman GL, Courtney KD, Andres V, Featherstone RM (1961). A new and rapid colorimetric determination of acetylcholinesterase activity. *Biochemical Pharmacology*.

[37] Laxton AW, Tang-Wai DF, McAndrews MP (2010). A phase I trial of deep brain stimulation of memory circuits in Alzheimer's disease. *Annals of Neurology*.

[38] Waite JJ, Chen AD, Wardlow ML, Wiley RG, Lappi DA, Thal LJ (1995). 192 Immunoglobulin G-saporin produces graded behavioral and biochemical changes accompanying the loss of cholinergic neurons of the basal forebrain and cerebellar Purkinje cells. *Neuroscience*.

[39] Fukutani Y, Cairns NJ, Rossor MN, Lantos PL (1996). Purkinje cell loss and astrocytosis in the cerebellum in familial and sporadic Alzheimer's disease. *Neuroscience Letters*.

[40] Sjöbeck M, Englund E (2001). Alzheimer's disease and the cerebellum: a morphologic study on neuronal and glial changes. *Dementia and Geriatric Cognitive Disorders*.

[41] Krnjevic K, Ropert N (1982). Electrophysiological and pharmacological characteristics of facilitation of hippocampal population spikes by stimulation of the medial septum. *Neuroscience*.

[42] Bilkey DK, Goddard GV (1985). Medial septal facilitation of hippocampal granule cell activity is mediated by inhibition of inhibitory interneurones. *Brain Research*.

[43] Ovsepian SV (2006). Enhancement of the synchronized firing of CA1 pyramidal cells by medial septum preconditioning: time-dependent involvement of muscarinic cholinoceptors and GABAB receptors. *Neuroscience Letters*.

[44] Winson J (1978). Loss of hippocampal theta rhythm results in spatial memory deficit in the rat. *Science*.

[45] Vertes RP, Kocsis B (1997). Brainstem-diencephalo-septohippocampal systems controlling the theta rhythm of the hippocampus. *Neuroscience*.

[46] Berry SD, Seager MA (2001). Hippocampal theta oscillations and classical conditioning. *Neurobiology of Learning and Memory*.

[47] Keita MS, Frankel-Kohn L, Bertrand N, Lecanu L, Monmaur P (2000). Acetylcholine release in the hippocampus of the urethane anaesthetised rat positively correlates with both peak theta frequency and relative power in the theta band. *Brain Research*.

[48] Yoder RM, Pang KCH (2005). Involvement of GABAergic and cholinergic medial septal neurons in hippocampal theta rhythm. *Hippocampus*.

[49] Barry C, Heys JG, Hasselmo ME (2012). Possible role of acetylcholine in regulating spatial novelty effects on theta rhythm and grid cells. *Frontiers in Neural Circuits*.

[50] Jessberger S, Clark RE, Broadbent NJ (2009). Dentate gyrus-specific knockdown of adult neurogenesis impairs spatial and object recognition memory in adult rats. *Learning and Memory*.

[51] Winocur G, Wojtowicz JM, Sekeres M, Snyder JS, Wang S (2006). Inhibition of neurogenesis interferes with hippocampus-dependent memory function. *Hippocampus*.

[52] Saxe MD, Battaglia F, Wang J (2006). Ablation of hippocampal neurogenesis impairs contextual fear conditioning and synaptic plasticity in the dentate gyrus. *Proceedings of the National Academy of Sciences of the United States of America*.

[53] Ming G, Song H (2005). Adult neurogenesis in the mammalian central nervous system. *Annual Review of Neuroscience*.

[54] Brown JP, Couillard-Després S, Cooper-Kuhn CM, Winkler J, Aigner L, Kuhn HG (2003). Transient Expression of Doublecortin during Adult Neurogenesis. *Journal of Comparative Neurology*.

[55] von Bohlen Und Halbach O (2007). Immunohistological markers for staging neurogenesis in adult hippocampus. *Cell and Tissue Research*.

[56] Couillard-Despres S, Winner B, Schaubeck S (2005). Doublecortin expression levels in adult brain reflect neurogenesis. *European Journal of Neuroscience*.

[57] Cooper-Kuhn CM, Winkler J, Kuhn HG (2004). Decreased neurogenesis after cholinergic forebrain lesion in the adult rat. *Journal of Neuroscience Research*.

[58] van der Borght K, Mulder J, Keijser JN, Eggen BJL, Luiten PGM, van der Zee EA (2005). Input from the medial septum regulates adult hippocampal neurogenesis. *Brain Research Bulletin*.

[59] Mohapel P, Leanza G, Kokaia M, Lindvall O (2005). Forebrain acetylcholine regulates adult hippocampal neurogenesis and learning. *Neurobiology of Aging*.

[60] Itou Y, Nochi R, Kuribayashi H, Saito Y, Hisatsune T (2011). Cholinergic activation of hippocampal neural stem cells in aged dentate gyrus. *Hippocampus*.

[61] Kaneko N, Okano H, Sawamoto K (2006). Role of the cholinergic system in regulating survival of newborn neurons in the adult mouse dentate gyrus and olfactory bulb. *Genes to Cells*.

[62] Tykocki T, Nauman P, Koziara H, Mandat T (2013). Microlesion effect as a predictor of the effectiveness of subthalamic deep brain stimulation for Parkinson's disease. *Stereotactic and Functional Neurosurgery*.

[63] Hamani C, Schwalb JM, Rezai AR, Dostrovsky JO, Davis KD, Lozano AM (2006). Deep brain stimulation for chronic neuropathic pain: Long-term outcome and the incidence of insertional effect. *Pain*.

[64] Pourfar M, Tang C, Lin T, Dhawan V, Kaplitt MG, Eidelberg DD (2009). Assessing the microlesion effect of subthalamic deep brain stimulation surgery with FDG PET. *Journal of Neurosurgery*.

[65] Robinson N, Duncan P, Gehrt M, Sancres A, Evans S (1975). Histochemistry of trauma after electrode implantation and stimulation in the hippocampus. *Archives of Neurology*.

[66] Hirshler YK, Polat U, Biegon A (2010). Intracranial electrode implantation produces regional neuroinflammation and memory deficits in rats. *Experimental Neurology*.

